# Comparative Analysis of Bacillariophyceae Chloroplast Genomes Uncovers Extensive Genome Rearrangements Associated with Speciation

**DOI:** 10.3390/ijerph191610024

**Published:** 2022-08-14

**Authors:** Yichao Wang, Jing Wang, Yang Chen, Shuya Liu, Yongfang Zhao, Nansheng Chen

**Affiliations:** 1 CAS Key Laboratory of Marine Ecology and Environmental Sciences, Institute of Oceanology, Chinese Academy of Sciences, Qingdao 266071, China; 2 Laboratory of Marine Ecology and Environmental Science, Qingdao National Laboratory for Marine Science and Technology, Qingdao 266237, China; 3 University of Chinese Academy of Sciences, Beijing 100049, China; 4 Center for Ocean Mega-Science, Chinese Academy of Sciences, Qingdao 266071, China; 5 Jiaozhou Bay National Marine Ecosystem Research Station, Institute of Oceanology, Chinese Academy of Sciences, Qingdao 266071, China; 6 Department of Molecular Biology and Biochemistry, Simon Fraser University, 8888 University Drive, Burnaby, BC V5A 1S6, Canada

**Keywords:** diatom, class Bacillariophyceae, chloroplast genome, phylogenetic analysis, super barcode

## Abstract

The Bacillariophyceae is a species-rich, ecologically significant class of Bacillariophyta. Despite their critical importance in marine ecosystems as primary producers and in the development of harmful algal blooms (HABs), taxonomic research on Bacillariophyceae species has been hindered because of their limited morphological features, plasticity of morphologies, and the low resolution of common molecular markers. Hence molecular markers with improved resolution are urgently needed. Organelle genomes, which can be constructed efficiently with the recent development of high throughput DNA sequencing technologies and the advancement of bioinformatics tools, have been proposed as super barcodes for their higher resolution for distinguishing different species and intra-species genomic variations. In this study, we tested the value of full-length chloroplast genomes (cpDNAs) as super barcodes for distinguishing diatom species, by constructing cpDNAs of 11 strains of the class Bacillariophyceae, including *Nitzschia ovalis*, *Nitzschia traheaformis*, *Cylindrotheca* spp., *Psammodictyon constrictum*, *Bacillaria paxillifer*, two strains of *Haslea tsukamotoi*, *Haslea avium*, *Navicula arenaria*, and *Pleurosigma* sp. Comparative analysis of cpDNAs revealed that cpDNAs were not only adequate for resolving different species, but also for enabling recognition of high levels of genome rearrangements between cpDNAs of different species, especially for species of the genera *Nitzschia*, *Cylindrotheca*, *Navicula* and *Haslea*. Additionally, comparative analysis suggested that the positioning of species in the genus *Haslea* should be transferred to the genus *Navicula*. Chloroplast genome-based evolutionary analysis suggested that the Bacillariophyceae species first appeared during the Cretaceous period and the diversity of species rose after the mass extinction about 65 Mya. This study highlighted the value of cpDNAs in research on the biodiversity and evolution of Bacillariophyceae species, and, with the construction of more cpDNAs representing additional genera, deeper insight into the biodiversity and evolutionary relationships of Bacillariophyceae species will be gained.

## 1. Introduction

Diatoms (Bacillariophyta) constitute a major group of phytoplankton as free-living organisms in both marine and freshwater environments and play important roles in biogeochemical cycles and the functioning of aquatic food webs [1,2,3,4,5]. Diatoms play an essential part in the cycling of CO_2_, which has been proposed to be comparable to that of all terrestrial rainforests combined in global carbon cycling and to contribute to approximately 20% of the total primary production on Earth [4,6,7,8]. As diatoms are frequently found in subfossil and fossil records because their unique silicified cell walls are resistant to decay, fossil records of diatoms are often used as a source of information for phylogenetic reconstruction and archeological research [9].

The Bacillariophyceae is a species-rich, ecologically diverse class of diatoms—species in this class are ubiquitous and can be found from polar regions to the tropics [10]. The biological significance of Bacillariophyceae in the biosphere is significant for many species. For example, *Nitzschia palea* has been identified as a promising candidate for economic production of biodiesel because of its prolific biomass production and high intracellular lipid content [11,12]. Several *Nitzschia* species have been used as indicators of heavy metal contamination and have been applied in water quality monitoring [13,14,15]. *Fragilariopsis kerguelensis* contributes most of the vast deposits of biogenic silica beneath the Southern Ocean (Atlantic sector) and provides information for research concerning the ancient environment [16]. Some Bacillariaceae species, especially many *Pseudo-nitzschia* species, produce domoic acid, a toxin that causes amnesic shellfish poisoning and presents serious environmental toxicology threats [17,18]. *Navicula* species have been found to improve the settlement of *Haliotis asinina* larvae and contribute to faster growth [19]. A hexane extract of *Navicula arenaria* is used as a biological control for diseases caused by phytopathogenic fungi [20]. *Haslea ostrearia* can produce a water-soluble blue pigment which is responsible for the greening of oyster’s gills and enhances their value to the oyster industry [21]. Furthermore, this blue pigment has biological functions, including antioxidant, antibacterial and antiviral properties [22,23]. *Fistulifera solaris* has a high neutral lipid content and growth rate, and is beneficial for biodiesel production [24].

Despite their critical importance, taxonomic research on the species of Bacillariophyceae has been hindered by the small cell sizes and simple morphologies of these species, which make them difficult to observe and study. In addition, the number of species in the Bacillariophyceae is high, containing many hundreds of species, including many species in the orders Bacillariales and Naviculales. Nevertheless, researchers have sought to study Bacillariophyceae species using both morphology-based methods and molecular marker-based methods. Trobajo et al. made an attempt to clarify the taxonomy of *Nitzschia* species using morphology-based analysis [25]. Several phylogenetic studies of the Bacillariophyceae have been undertaken using the common molecular marker 18S rDNA [26,27]. These studies have improved our knowledge of Bacillariophyceae species. Despite these efforts, the results from phylogenetic studies using common molecular markers contained nodes with weak or no statistical support, suggesting that these common molecular markers are inadequate to represent genetic diversity.

Chloroplast genomes (cpDNAs) have been proposed to be “super barcodes” that have much higher resolution for distinguishing species for their longer sequence lengths than common molecular markers, and have been increasingly used in evolutionary studies [28,29,30]. Complete cpDNA sequences have also been widely used for evolutionary biology and ecological applications [31,32]. In addition, chloroplast genome sequences have revealed considerable variation within and between plant species in terms of both sequence and structural variation. Genome rearrangements influence gene order and configuration of gene clusters in all genomes [33]. Chloroplast genomes in most land plants share a highly conserved gene content and similar gene orders. However, the diversity of diatom cpDNA is sometimes high [34]. Driven by the development of DNA sequencing technologies and the advancement of bioinformatics analysis algorithms, more cpDNAs of species in Bacillariophyta have been fully constructed which help to provide valuable knowledge about phylogenetic relationships and evolutionary trajectories [35,36,37,38]. Nevertheless, the study of genome rearrangements and the speciation of Bacillariophyceae species has been limited.

In this study, we reported 11 complete cpDNAs for species in the class Bacillariophyceae including *Nitzschia ovalis*, *Nitzschia traheaformis*, two strains of *Cylindrotheca* sp., *Psammodictyon constrictum*, *Bcillaria paxillifer*, two strains of *Haslea tsukamotoi*, *Haslea avium*, *Navicula arenaria* and *Pleurosigma* sp. Comparative analysis of these cpDNAs revealed extensive genomic structural differences, including gene loss, gene duplication, and genome rearrangements. Furthermore, we also evaluated the speciation of Bacillariophyceae species.

## 2. Materials and Methods

### 2.1. Strain Isolation and Culturing

Eleven diatom strains studied in this project (CNS00113, CNS00381, CNS00383, CNS00409, CNS00433, CNS00435, CNS00419, CNS00432, CNS00319, CNS00326 and CNS00413) were isolated from water samples collected in Jiaozhou Bay, onboard the R/V Chuangxin, which was operated by the Jiaozhou Bay Marine Ecosystem Research Station, using single-cell capillary methods. Cells were cultured in L1 medium with 1‰ volume fraction Na_2_SiO_3_ with H_2_O added. The culture temperature was 18–20 °C and the illuminance was from 2000 Lux to 3000 Lux for a photoperiod of 12 h light–12 h dark, as previously described [39].

### 2.2. DNA Library Preparation and Whole Genome Sequencing

Total DNA was extracted using the DNAsecure Plant Kit (Tiangen Biotech, randomized controlled trial, Beijing, China) as previously described [39]. Genomic DNA samples were fragmented by sonication (Covaris S220, Covaris, Woburn, MA, USA) to a size of 350 bp. DNA fragments were then end-polished, A-tailed, and ligated with the full-length adapters for Illumina sequencing, followed by PCR (MiniAmp Thermal Cycler, ThermoFisher, Waltham, MA, USA) enrichment using generic adapter P5 and P7 oligos. The DNA libraries were sequenced using NovaSeq PE150 (Illumina, San Diego, CA, USA) and paired-end reads of 150 bp size were generated.

### 2.3. Construction of Molecular Marker

A full-length sequence of the common molecular marker 18S rDNA was constructed for each strain using Illumina sequencing results. Full-length 18S rDNA-based phylogenetic trees were constructed using MEGA7 [40]. Phylogenetic relationships were inferred using the maximum likelihood method. The percentage of replicate trees in which the associated taxa clustered together in the bootstrap test (1000 replicates) was shown next to the branches [41].

### 2.4. Construction and Annotation of cpDNAs

Illumina sequencing raw data were trimmed using a Trimmomatic-0.39 read-trimming tool with the parameters: LEADING:3 TRAILING:3 SLIDING WINDOW:4:15 MINLEN:75 [42]. Clean reads were assembled to obtain complete cpDNAs using GetOrganelle-1.7.4.1 [43]. Verification of cpDNA sequences was accomplished by aligning Illumina reads against cpDNAs using BWA-0.7.17 [44] and SAMtools-1.10 [45], which was visualized using IGV-2.7.2 [46]. Annotation was conducted with MFannot (https://github.com/BFL-lab/Mfannot (accessed on 15 December 2021)) and NCBI’s ORF Finder (https://www.ncbi.nlm.nih.gov/orffinder/ (accessed on 15 January 2022)), which was further completed using NCBI’s Sequin15.10 (https://www.ncbi.nlm.nih.gov/projects/Sequin/ (accessed on 15 January 2022)). For accuracy of comparative analysis, we inspected and re-annotated all cpDNAs that were downloaded from the NCBI.

### 2.5. Phylogenetic Analysis of cpDNAs

Amino acid sequences of 108 protein-coding genes (PCGs) from the cpDNA of each diatom species studied in this project were extracted and concatenated for phylogenetic analysis (Table 1). The amino acid sequences of the 108 genes were individually aligned using MAFFT [47]. Ambiguously aligned regions were trimmed by trimAl 1.2rev59 [48], and then concatenated using PhyloSuite [49]. The best-fit model was identified using ModelFinder [50]. A phylogenetic tree was constructed with IQ-TREE [51]. An ultrafast bootstrap approximation was conducted with 5000 replicates of the dataset [52]. *Triparma laevis* was used as the outgroup.

### 2.6. Synteny Analysis

Synteny analysis of cpDNAs was carried out using the progressiveMauve program of the Mauve v2.3.1 package [53]. Comparative illustration of cpDNAs was performed using circos-0.69 [54].

### 2.7. Divergence Time Estimation

Phylogenetic relationships and molecular dating were analyzed by calculating the codon evolution rate of the nucleotide sequences of the protein-coding genes (PCGs). The nucleotide sequences were aligned using MAFFT with Codon and concatenated in the software PhyloSuite [49]. A phylogenetic tree was constructed using IQ-TREE [51]. Molecular dating was conducted using the PAML package [55]. Briefly, a rough estimation of the substitution rate was performed using baseml, and estimation of divergence times with the approximate likelihood method was performed using mcmctree. The phylogenetic tree was displayed in Figtree and visualized with a 95% highest posterior density interval (HPD) for each node. The *Ectocarpus siliculosus* was used as the outgroup with its known fossil time. Three calibrations of the internal nodes in divergence time estimation were performed for *Ectocarpus siliculosus* 176–202 Mya, *Fragilariopsis kerguelensis* 10–36 Mya [56] and *Rhizosolenia setigera* 90–93 Mya [57].

## 3. Results

### 3.1. Molecular and Morphological Characterization of 11 Diatom Strains

**Figure 1:**
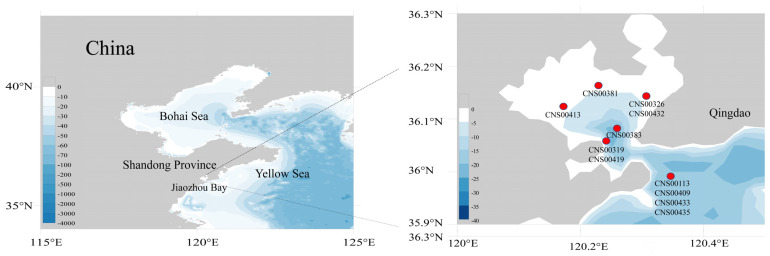
Sampling sites of 11 diatom species in Jiaozhou Bay.

**Figure 2:**
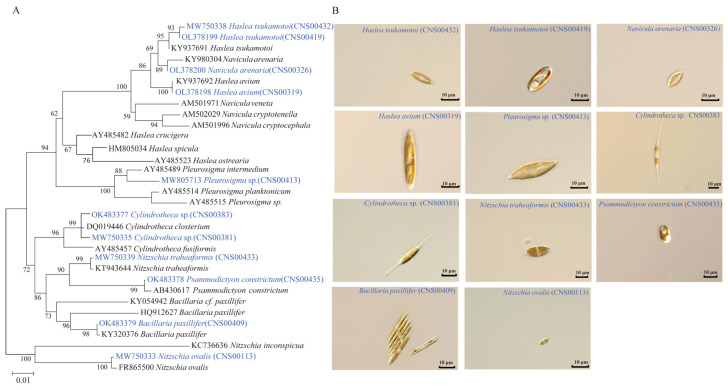
Morphological and molecular analysis of diatom species analyzed in this study. (**A**) Phylogenetic analysis based on 18S ribosomal DNA (18S rDNA) gene. Numbers at the branches represent bootstrap values. Branch lengths are proportional to the genetic distances, which are indicated by the scale bar. (**B**) Representative micrographs of 11 diatom species studied in this project. Bar = 10 μm.

The eleven diatom strains of the class Bacillariophyceae analyzed in this study were all isolated from Jiaozhou Bay, China (Figure 1). These strains were annotated using both morphological features and DNA sequences of the molecular marker 18S rDNA, with percentage identity (PID) > 99% to corresponding reference sequences (Figure 2A). The morphologies of two strains (CNS00113 and CNS00433) shared similarities with *Nitzschia* species [25]. Both were unicellular and their cells were long, straight, and ovoid (Figure 2B). The strain CNS00113 was further annotated as *Nitzschia ovalis* based on the similarity of its full-length 18S rDNA (MW750333) to that of *Nitzschia ovalis* (FR865500), with the percentage identity (PID) being 99.82% [58]. Similarly, the strain CNS00433 was annotated as *Nitzschia traheaformis* based on the similarity of its full-length 18S rDNA (MW750339) to that of *Nitzschia* *traheaformis* (KT943644), with the PID being 99.69% (Figure 2A) [59]. The strains CNS00381 and CNS00383 were unicellular and long with a cylindrical central part and elongated ends and their morphologies resembled *Cylindrotheca closterium* (Figure 2B). In spite of the similarity of their full-length 18S rDNA sequences (MW750335 and OK483377, respectively) to that of *Cylindrotheca closterium* (DQ019446) (99.71% and 99.77%, respectively) [60], they were annotated as *Cylindrotheca* spp. The strain CNS00435, whose cells were unicellular and had a slight middle constriction, was annotated as *Psammodictyon constrictum* (99.77% PID with AB430617) [61]. The strain CNS00409, whose cells were stick-shaped and able to connect with adjacent cells into a chain that can slide, was annotated as *Bacillaria paxillifer* (99.94% PID with KY320376) [62]. The cells of CNS00319, CNS00326, CNS00419, CNS00432 and CNS00413 were all spindle-like, which is a feature shared by species of the order Naviculales. In particular, the strains CNS00326, CNS00419, and CNS00432 all had a ‘sandwich-type’ valve structure (Figure 2B). The strain CNS00326 was further annotated as *Navicula arenaria* because its 18S rDNA sequence showed 99.76% PID with KY980304. Similarly, the strains CNS00419 and CNS00432 were all annotated as *Haslea tsukamotoi* (99.70% and 99.61% PID with KY937691, respectively), while the strain CNS00319 was annotated as *Haslea avium* (99.94% PID with KY937692) [63]. The strain CNS00413 was annotated as *Pleurosigma* sp. (98.31% PID with AY485514) [57].

### 3.2. Construction and Comparative Analysis of cpDNAs

We successfully constructed complete cpDNAs for 11 diatom strains, in which six belonged to the family Bacillariaceae, four belonged to the family Naviculaceae, and one belonged to the family Pleurosigma (Table 2). The genome sizes of 11 cpDNAs varied substantially, ranging from 119,499 bp (OK505012 *Bacillaria paxillifer*) to 188,592 bp (OK505009 *Cylindrotheca* sp.). The AT contents ranged from 68.6% (OL415007 *Haslea tsukamotoi* and OL415008 *Haslea tsukamotoi*) to 70.4% (OL415008 *Pleurosigma* sp.). Each cpDNA had typical four conjoined structures with one long single copy (LSC) (ranging in size from 63,955 bp in OK505012 *Bacillaria paxillifer* to 98,331 bp in OK505009 *Cylindrotheca closterium*), one short single copy (SSC) (ranging in size from 41,892 bp in OK505012 *Bacillaria paxillifer* to 73,153 bp in OK505011 of *Psammodictyon constrictum*), and two inverted repeats (IRs) of identical sizes (ranging in size from 7011 bp in OK505012 *Bacillaria paxillifer* to 15,462 bp in OK505010 *Cylindrotheca* sp.) except for KR709240 Pseudo-nitzschia multiserie which had no reported inverted repeats [64]. All 11 of these cpDNAs contained six non-coding rRNA genes (two copies of each of the three genes *rns*, *rnl*, *rrn5*). The number of PCGs ranged from 121 (OK505008 *Pleurosigma* sp.) to 137 (OK505009 *Cylindrotheca* sp.).

**Figure 3:**
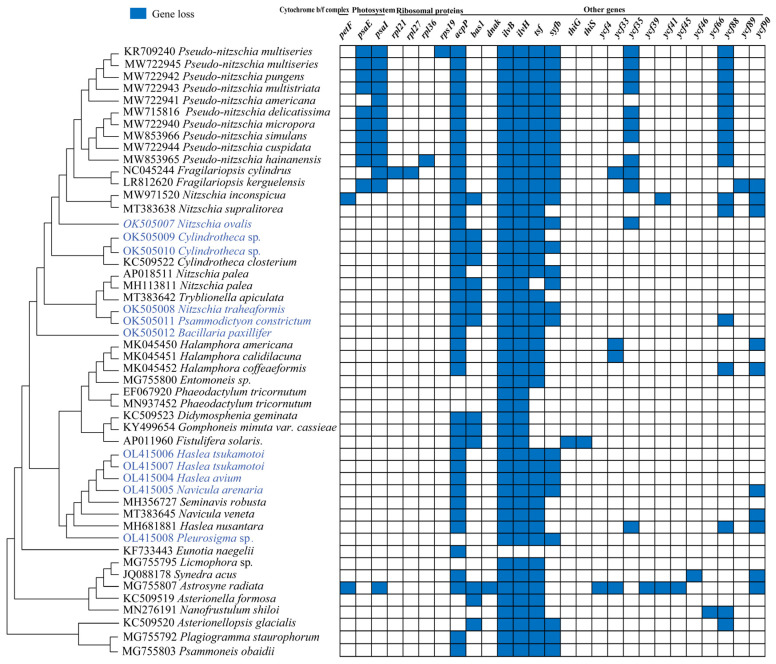
Gene losses in diatom cpDNAs. The matrix shows the presence (empty) and absence (blue) of 27 genes among the cpDNAs of Bacillariophyceae.

Gene loss in cpDNAs in diatom species was frequent [35]. Multiple cases of independent gene loss events were identified in the cpDNAs of the class Bacillariophyceae (Figure 3). Of the genes of the cytochrome b/f complex, only *petF* was found missing from the cpDNA of *Nitzschia inconspicua* and *Astrosyne radiate*; of the photosystem genes, *psaE* was missing from all *Pseudo-nitzschia* cpDNAs except from the *Pseudo-nitzschia americana* cpDNA and from the *Fragilariopsis kerguelensis* cpDNA. Similarly, the photosystem gene *psaI* was missing from all *Pseudo-nitzschia*, *Fragilariopsis* and *Astrosyne radiata* cpDNAs. Of the ribosomal protein genes, *rpl21* and *rpl27* were found missing from *Fragilariopsis cylindrus*, *rpl36* and *rps19* were found missing from the cpDNAs of only one species, including *Pseudo-nitzschia hainanensis* and *Pseudo-nitzschia multiseries*, respectively. Of the other genes, many genes were found missing in different species, suggesting that they were the least conserved genes in the evolution of navicular chloroplasts.

### 3.3. Plasticity of the Inverted Repeat Regions in Bacillariophyceae

**Figure 4:**
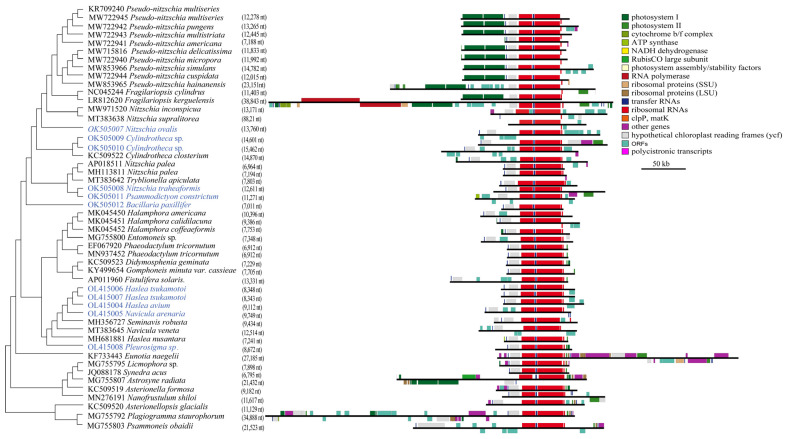
Size variations of the inverted repeated region across cpDNAs of Bacillariophyceae. Colored boxes circumscribe genes in various functional categories, with those above the line transcribed on the forward strand and vice versa for genes below the line.

The inverted repeat (IR) region in Bacillariophyceae varies in length by nearly five-fold, from ~7 kb in *Bacillaria paxillifer* to ~38 kb in *Fragilariopsis kerguelensis* (Figure 4). All IR regions contained a conservative gene block (*rns*-*trnI*-*trnA*-*rnl*-*rrn5*) except for that of *Nitzschia inconspicua*, which contained an extra-unidentified gene *orf134* inserted into this gene cluster (*rns*-*orf134*-*trnI*-*trnA*-*rnl*-*rrn5*). In some cases, IR expansions have resulted in the duplication of a large number of genes. The IR expansion of *Fragilariopsis kerguelensis* resulted in the duplication of over a dozen PCGs (Figure 4).

### 3.4. Phylogenetic Analysis of the Bacillariophyceae

**Figure 5:**
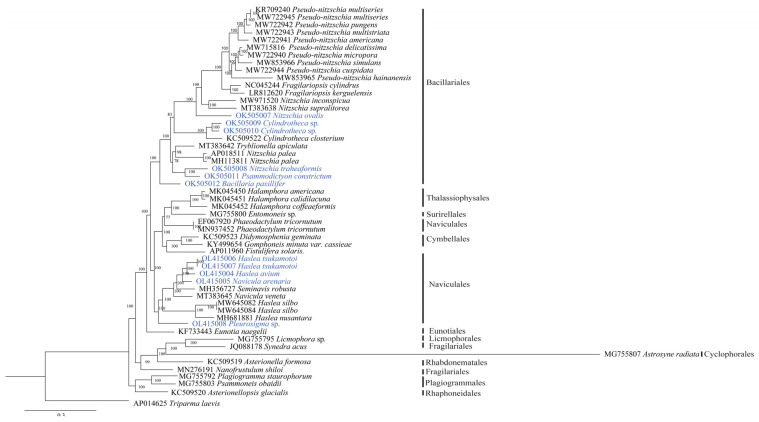
Maximum likelihood (ML) phylogenetic tree of Bacillariophyceae species based on concatenated amino acid sequences encoded by 108 shared protein-coding genes. Numbers on the left- and right-side at the branches represent bootstrap values. Branch lengths were proportional to the amount of sequence change, which are indicated by the scale bar below the trees.

To explore the evolutionary relationships between the 11 cpDNAs newly constructed in this project and those of class Bacillariophyceae diatom species reported previously, we constructed a phylogenetic tree using the amino acid sequences of PCGs shared by these cpDNAs using a maximum likelihood method (Figure 5). The phylogenetic analysis suggested that the positioning of many species might need revision. In the order Bacillariales, the cpDNAs of *Nitzschia ovalis*, *Nitzschia traheaformis* did not cluster with cpDNAs of other species annotated in the genus *Nitzschia* in the phylogenetic tree. In contrast, *Nitzschia inconspicua* [65] and *Nitzschia supralitorea* [66] formed a clade that did not cluster with other *Nitzschia* species, suggesting that the genus *Nitzschia* may need to be split into multiple genera. *Cylindrotheca* sp. (OK505009 of the strain CNS00381) and *Cylindrotheca* sp. (OK505010 of the strain CNS00383) clustered with another strain annotated as *Cylindrotheca closterium* (KC509522) [67]. *Psammodictyon constrictum* was found to group closely with *Nitzschia traheaformis*. *Bacillaria paxillifer* formed an independent clade.

The five cpDNAs constructed in this study of the Naviculales included three cpDNAs (*Haslea tsukamotoi* OL415006, *Haslea tsukamotoi* OL415007, and *Haslea avium* OL415004) of the genus *Haslea*, one cpDNA (*Navicula arenaria* OL415005) of the genus *Navicula*, and one cpDNA (*Pleurosigma* sp. OL415008) of the genus *Pleurosigma* (Figure 5). Interestingly, the cpDNAs of *Haslea tsukamotoi* and *Haslea avium* were close to *Navicula arenaria*, rather than with the cpDNAs of *Haslea silbo* and *Haslea nusantara* in the genus *Haslea*, suggesting that the positioning of these *Haslea* species may need revisiting. A single species *Pleurosigma* sp. was found in the genus *Pleurosigma*, suggesting that additional sampling of species in many genera, including this one, is needed.

### 3.5. Inter- and Intra-Genus Synteny Analysis of cpDNAs

**Figure 6:**
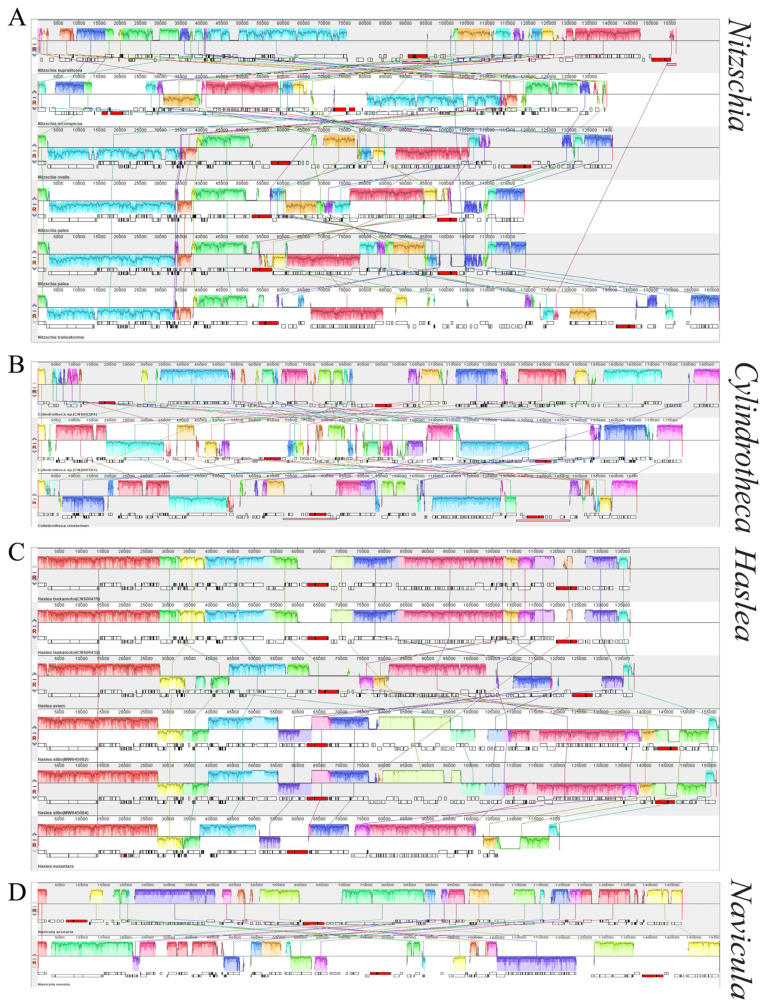
Synteny analysis of cpDNAs. (**A**) Synteny analysis of *Nitzschia* cpDNAs (**B**) Synteny analysis of *Cylindrotheca* cpDNAs. (**C**) Synteny analysis of *Haslea* cpDNAs. (**D**) Synteny analysis of *Navicula* cpDNAs. Each colored block indicates a synteny block among different cpDNAs.

**Figure 7:**
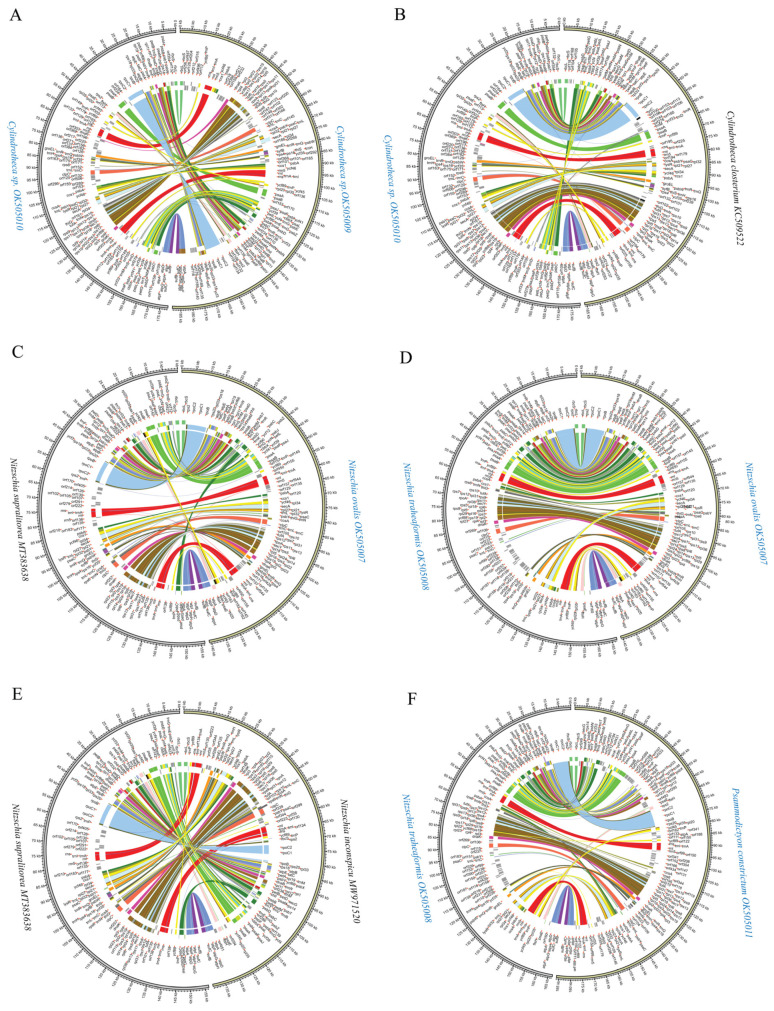
Pair-wise comparison of representative Bacillariales cpDNAs. (**A**) The comparative analysis of *Cylindrotheca* sp. OK505009 and *Cylindrotheca* sp. OK505010 cpDNAs. (**B**) The comparative analysis of *Cylindrotheca closterium* KC509522 and *Cylindrotheca* sp. OK505010 cpDNAs. (**C**) The comparative analysis of *Nitzschia ovalis* OK505007 and *Nitzschia supralitorea* MT383638 cpDNAs. (**D**) The comparative analysis of *Nitzschia ovalis* OK505007 and *Nitzschia traheaformis* OK505008 cpDNAs. (**E**) The comparative analysis of *Nitzschia inconspicu* MW971520 and *Nitzschia supralitorea* MT383638 cpDNAs. (**F**) The comparative analysis of *Psammodictyon constrictum* OK505011 and *Nitzschia traheaformis* OK505008 cpDNAs. The assignment of genes into different functional groups is indicated by colors.

Of species in the genus *Nitzschia*, two cpDNAs constructed in this study, and four previously reported cpDNAs, exhibited highly diverse cpDNA genome arrangements (Figure 6A). Pair-wise analysis of the cpDNAs of *Nitzschia ovalis* and *Nitzschia supralitorea*, and *Nitzschia ovalis* and *Nitzschia traheaformis*, showed extensive differences in gene arrangements of cpDNAs (Figure 7C,D). The cpDNAs of *Nitzschia inconspicua* and *Nitzschia supralitorea*, which clustered with each other in the phylogenetic analysis of PCGs, also exhibited a high level of genome arrangements in the cpDNAs (Figure 7E).

Extensive genome rearrangements were also found between cpDNAs in *Cylindrotheca*. The chloroplast genomes of two *Cylindrotheca* sp. strains (CNS00381 and CNS00383) constructed in this study, and one reported cpDNA of *Cylindrotheca closterium* (DQ019446), showed high levels of genome rearrangements (Figure 6B). Gene-level analysis revealed that extensive genome rearrangements were identified in the cpDNAs of three *Cylindrotheca closterium* strains, two of which were identified in this project (Figure 7A,B), suggesting that these strains may actually represent unique species. Highly diverse genome arrangements were found in the cpDNAs of *Psammodictyon constrictum* and *Nitzschia traheaformis* (Figure 7F).

**Figure 8:**
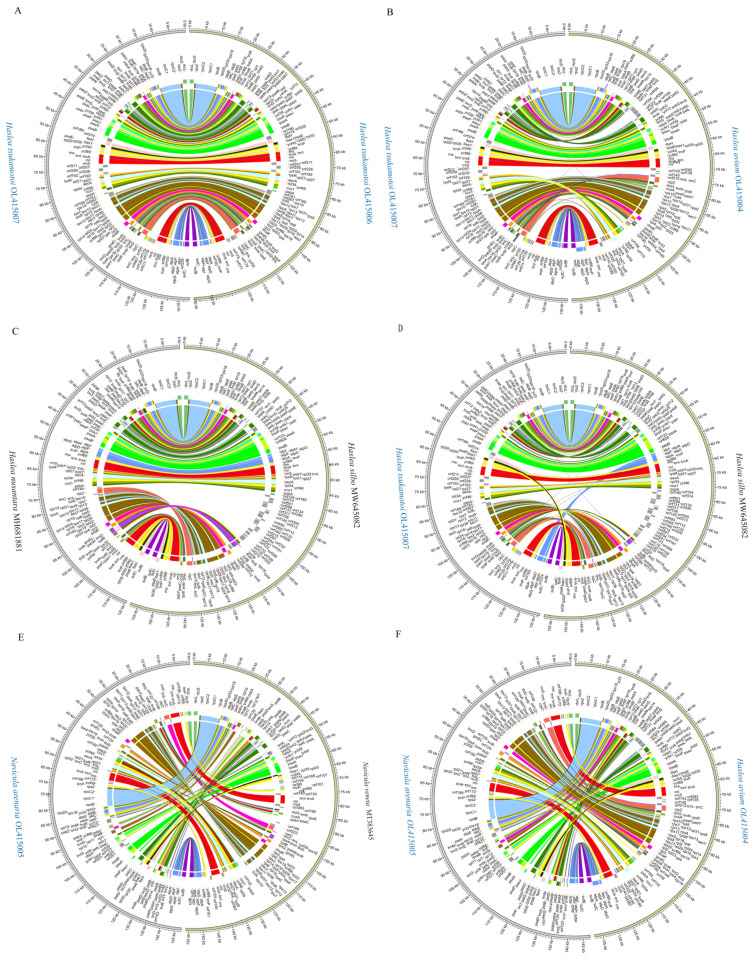
Pair-wise comparison of representative Naviculales cpDNAs. (**A**) The comparative analysis of *Haslea tsukamotoi* OK415006 and *Haslea tsukamotoi* OK415007 cpDNAs. (**B**) The comparative analysis of *Haslea avium* OK415004 and *Haslea tsukamotoi* OK415007 cpDNAs. (**C**) The comparative analysis of *Haslea silbo* MW646082 *and Haslea nusantara* MH681881 cpDNAs. (**D**) The comparative analysis of *Haslea silbo* MW646082 *and Haslea tsukamotoi* OK415007 cpDNAs. (**E**) The comparative analysis of *Navicula arenaria* OK415005 *and Navicula veneta* MT383645cpDNAs. (**F**) The comparative analysis of *Navicula arenaria* OK415005 and *Haslea avium* OK415004 cpDNAs. The assignment of genes into different functional groups is indicated by colors.

Of species in the genera *Haslea* and *Navicula*, three cpDNAs and one cpDNA constructed in this study, and three and one previously reported cpDNAs, exhibited highly diverse cpDNA genome arrangements, respectively (Figure 6C,D). Pair-wise analysis of cpDNAs of two newly constructed *Haslea tsukamotoi* cpDNAs showed conservative gene arrangements (Figure 8A). Pair-wise analysis of cpDNAs of *Haslea tsukamotoi and Haslea acium*, *Haslea silbo* and *Haslea nusantara*, and *Haslea silbo* and *Haslea tsukamotoi* showed extensive differences in the gene arrangements of cpDNAs (Figure 8B–D). The cpDNAs of *Navicula arenaria* and *Navicula veneta* exhibited a high level of genome arrangements in cpDNAs (Figure 8E). The cpDNAs of *Navicula arenaria* and *Haslea avium* were close in the phylogenetic analysis of PCGs but exhibited a high level of genome arrangements in cpDNAs (Figure 8F). These analyses suggested that the genome structures of cpDNAs of species in the family Naviculaceae, including the genera *Haslea* and *Navicula*, showed high levels of genome rearrangements.

### 3.6. Divergence Time Estimation of Bacillariophyceae Species

**Figure 9:**
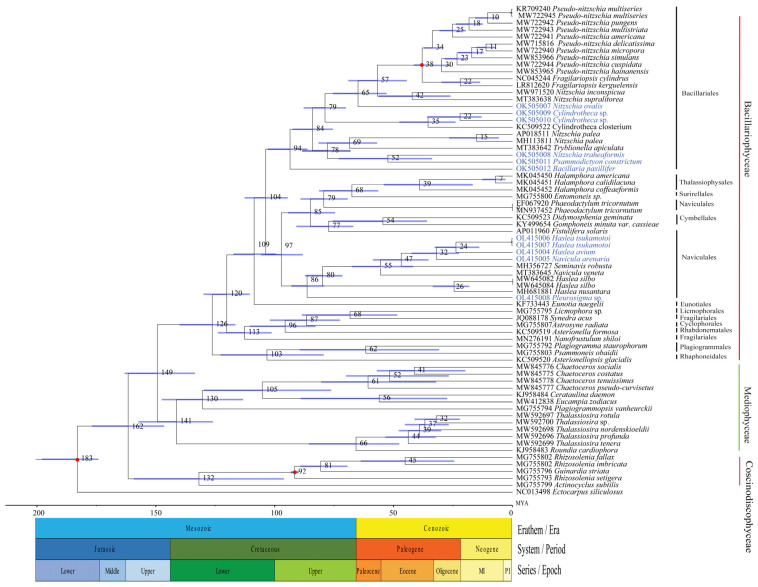
Time-calibrated phylogeny of cpDNAs of Bacillariophyceae species. The red dots represent calibration point and the 95% highest posterior density interval for node ages are shown with translucent blue bars.

To explore the divergence time between diatom species in the class Bacillariophyceae, we constructed a molecular dating tree (Figure 9). The class Bacillariophyceae evolved out at 149 Mya (95% HPD: 163–134 Mya). In the order Bacillariales, *Bacillaria paxillifer* from the genus *Bacillaria* was the first to evolve out at 94 Mya (95% HPD: 103–86 Mya). The divergence between the *Nitzschia traheaformis* and *Psammodictyon constrictum* occurred at 52 Mya (95% HPD: 73–34 Mya), and *Nitzschia ovalis* split at 65 Mya (95% HPD: 76–53 Mya). The evolution of genus *Cylindrotheca* occurred at 79 Mya (95% HPD: 88–70 Mya) in which *Cylindrotheca closterium* appeared at 35 Mya (95% HPD: 48–24 Mya). In the order Naviculales, the emergence of the genus *Pleurosigma* occurred at 86 Mya (95% HPD: 93–79 Mya). The genus *Haslea* appeared at 80 Mya (95% HPD: 87–72 Mya), and then *Haslea silbo* and *Haslea nusantara* emerged at 26 Mya (95% HPD: 37–16 Mya), almost during the same period, the divergence between *Haslea avium* and *Haslea tsukamotoi* occurred at 24 Mya (95% HPD: 33–14 Mya). The split between *Navicula arenaria* and *Haslea avium* occurred at 32 Mya (95% HPD: 42–22 Mya).

## 4. Discussion

### 4.1. Chloroplast Genomes Are Useful Super Barcodes

Although diatoms have been estimated to comprise 200,000 species, only about 10,000 species have been described and annotated [2,68]. This estimation was based on the assumption that diatom taxonomy was “too coarse-grained”, “combining different species under the same name”, and that there are “understudied habitats” [68]. Thus, the vast majority of diatom species remain to be identified. The proper taxonomy of these diatom species remains challenging partly because different diatom species may show high morphological similarity [69,70] and high morphological plasticity [9]. The application of methods and common molecular markers have facilitated diatom taxonomy. However, most common molecular markers, including 18S rDNA, 28S rDNA, ITS, *rbcL*, and *cox1* have limited resolution. Chloroplast genomes of the Bacillariaceae show a high level of differences (Figure 5), supporting the idea of using cpDNAs as super barcodes, consistent with the proposal of using cpDNAs as super barcodes for distinguishing different plant species [30].

### 4.2. Gene Loss and Plasticity of the IR Regions

The loss of important genes from cpDNAs has been previously reported in many species of Bacillariophyta [31,35], and for the cpDNAs of plant species [71]. Some *psa* genes are not essential for a complete and functional photosynthetic process and might be compensated for by other *psa* or *psb* genes or by a nuclear-encoded gene [72]. The gene *petF* transferring from cpDNAs to the nucleus was one of the mechanisms that enabled the oceanic diatom *Thalassiosira oceanica* to exhibit remarkable tolerance to low-iron conditions [37]. Further studies could help the determination of whether the *petF* of *Nitzschia inconspicua* [65] and *Astrosyne radiate* [31] cpDNAs transfers to the nuclear genome. The mechanisms that compensate for *psaE* and *psaI* loss in the genera *Pseudo-nitzschia* and *Fragilariopsis* and in *Astrosyne radiata* remain to be studied (Figure 3). The *bas1* was lost in many species of Bacillariophyceae and the loss occurred in both red algal and chromalveolate plastid genomes [73,74,75]. The gene *syfB* was one of the last remaining tRNA synthetized genes in primary and secondary red plastids [35], while *syfB* was lost in a large number of species of Bacillariaceae. The frequent loss of *ycf* genes, which encodes a hypothetical protein, indicates that they were the least conservative genes in the chloroplast evolution of Bacillariaceae species.

A typical cpDNAs genome structure consists of four main segments, referred to as the small single copy region (SSC), the large single copy region (LSC), and two inverted repeat regions (IR regions) [76,77]. The contraction and expansion of the IR region was one of the factors contributing to variations in the sizes of cpDNAs and resulted in a large number of gene duplications in diatoms [31,78]. Compared to the size of the IR region, ranging from 10 to 15 kb in non-seed plants to 20–30 kb in angiosperms [72,77,79,80], the size of the IR region, from 7 to 38 kb, in Bacillariaceae species indicated they had higher plasticity (Figure 4). Most notably, although the deletion of IR regions had been previously reported in a few plants [81,82], the lack of IR regions in the published cpDNA of KR709240 *Pseudo-nitzschia multiseries* was likely due to assembly errors because the cpDNA of another *Pseudo-nitzschia multiseries* strain, and the cpDNAs of eight species of the genus *Pseudo-nitzschia*, all had two IR regions.

### 4.3. Extensive Genome Arrangements between Bacillariophyceae Species

The genus *Nitzschia* exhibited highly diverse cpDNA genome arrangements, which was different from many diatom genera including *Thalassiosira*, *Chaetoceros*, and *Skeletonema*, in which the cpDNA genome exhibited high consistency [83,84,85]. The genus *Cylindrotheca* consisted of a small group of marine diatoms, with a few species described, including the existence of cryptic species, such as *Cylindrotheca closterium* [60,86,87]. Despite the two cpDNAs of *Cylindrotheca* sp. constructed in this study being close to other published *Cylindrotheca closterium* cpDNAs and the similarity of full-length 18S rDNA sequences to that of *Cylindrotheca closterium* (DQ019446) [60] being 99.71% and 99.77%, respectively, the degree of variation of the cpDNA genome arrangements was quite high, which suggested that there are new species in the genus *Cylindrotheca*.

*Bacillaria paxillifer* was the first to be separated into a genus of its own, *Bacillaria* [88]. *Bacillaria paxillifer* formed an independent branch in the phylogenetic analysis (Figure 5), which supported the division. The observation of *Psammodictyon constrictum* clustering with *Nitzschia traheaformis* was consistent with a previous study [10], while there were massive gene rearrangements of cpDNAs, suggesting that the relationship between the genera *Psammodictyon* and *Nitzschia* is more complicated than the current classification. Efforts to construct more cpDNAs of Bacillariaceae species will help greatly in the optimization of the current classification.

The genus *Haslea* is a morphologically highly diverse group [63]. In this study, the species of the genus *Haslea* in the phylogenetic analysis were divided into two separate clades that exhibited highly diverse cpDNA genome arrangements in the synteny analysis, which pointed to the possibility that the genus *Haslea* may be non-monophyletic. *Haslea tsukamotoi* and *Haslea avium* belonged to a clade that also contained *Navicula* species, suggesting that these two species may belong to the genus *Navicula*. This observation is consistent with a previously published study proposing the transfer of *Haslea tsukamotoi* and *Haslea avium* to the genus *Navicula* based on morphological data and molecular marker analysis [63]. There is another independent clade containing *Haslea nusantara* and *Haslea silbo*, both sharing the interesting trait of being capable of producing blue pigments [21,89]. Efforts to construct more cpDNAs of Naviculales species will help to strengthen the current classification.

### 4.4. The Origin and Speciation of Bacillariophyceae Species

Fossil evidence suggests that diatoms originated in the late Jurassic period and became more common in the mid-Cretaceous period [90,91,92,93]. In the light of many diatom deposits lost as a result of diagenesis, the comparative analysis of cpDNAs could provide valuable complementary genetic insights into diatom evolution. In this study, we used diatom cpDNAs as a “super barcode” to infer the time frame within which Bacillariophyceae species originated and diversified. Our study indicated that the species of Bacillariophyceae emerged during the Cretaceous period, which is consistent with the published time-calibrated analysis using two nuclear rRNA genes (18S and 28S rRNA genes), seven plastid genes (16S rRNA, *atpB*, *psaA*, *psaB*, *psbA*, *psbC* and *rbcL*) and two mitochondrial genes (*cob* and *cox1*) [94]. During this time frame, diatoms played a dominant role in the carbon cycle when O_2_ levels in the atmosphere were rising [95,96]. Most Bacillariophyceae species began to appear after the end of the Cretaceous period (66 Mya) and became prosperous during the Cenozoic period. The main reasons for this were, probably, (1) the mass extinction of about 65 Mya which led to the disappearance of about 85% of species, except diatoms, and offered ecological opportunities for the emergence of new species [97], and (2) throughout the Cenozoic period, the bioavailability of silica was rising because of increased weathering of silicate rock [98] and the nutrient-rich seawater brought by the Antarctic circumpolar current [99,100].

## 5. Conclusions

In this study, we constructed 11 complete chloroplast genomes of Bacillariophyceae species and analyzed them in a comparative framework with other published cpDNAs of Bacillariophyceae species. We demonstrated that cpDNAs could be used as super barcodes for distinguishing Bacillariophyceae species. Furthermore, we have also shown that comparative analysis of cpDNAs may reveal critical events in evolution, including gene losses. Some PCGs losses were found in the cpDNAs in Bacillariophyceae species and the size of the IR regions showed high plasticity. The relationships between the Bacillariophyceae species, especially species in the genus *Nitzschia* and the genus *Cylindrotheca*, in which there were many genome rearrangements among the cpDNAs, were complicated. The species *Cylindrotheca closterium* may represent many cryptic species with nearly identical full-length 18S rDNAs, but highly divergent cpDNAs. The Bacillariophyceae species evolved out during the Cretaceous period and the diversity of species began clearly to rise after the mass extinction about 65 Mya. With the construction of more cpDNAs of Bacillariophyceae species, more insight will be gained into the biodiversity and genetic evolutionary relationships of Bacillariophyceae species.

## Figures and Tables

**Table 1:** 108 shared protein-coding genes of cpDNAs in Bacillariophyceae.

Category	Genes
Photosystem I	*psaA*, *psaB*, *psaC*, *psaD*, *psaF*, *psaM*, *psaJ*, *psaL*
Photosystem II	*psbB*, *psbC*, *psbD*, *psbE*, *psbF*, *psbH*, *psbI*, *psbJ*, *psbK*, *psbL*, *psbN*, *psbT*, *psbV*, *psbX*, *psbY*, *psbZ*
Cytochrome b/f complex	*petA*, *petB*, *petD*, *petG*, *petL*, *petM*, *petN*
ATP synthase	*atpA*, *atpB*, *atpD*, *atpE*, *atpF*, *atpG*, *atpH*, *atpI*
RubisCO subunit	*rbcL*, *rbcS*
RNA polymerase	*rpoA*, *rpoB*, *rpoC1*, *rpoC2*
Ribosomal proteins (SSU)	*rps2*, *rps3*, *rps4*, *rps5*, *rps7*, *rps8*, *rps9*, *rps10*, *rps11*, *rps12*, *rps13*, *rps14*, *rps16*, *rps17*, *rps18*, *rps20*
Ribosomal proteins (LSU)	*rpl1*, *rpl2*, *rpl3*, *rpl4*, *rpl5*, *rpl6*, *rpl11*, *rpl12*, *rpl13*, *rpl14*, *rpl16*, *rpl18*, *rpl20*, *rpl23*, *rpl24*, *rpl29*, *rpl31*, *rpl32*, *rpl33*, *rpl34*, *rpl35*
Other genes	*cbbX*, *ccs1*, *ccsA*, *chlI*, *clpC*, *dnaB*, *dnaK*, *ftsH*, *groEL*, *lysR*, *secA*, *secG*, *secY*, *sufB*, *sufC*, *tatC*, *tufA*, *thiG*, *thiS*, *ycf3*, *ycf4*, *ycf12*, *ycf39*, *ycf41*, *ycf46*, *ycf66*

**Table 2:** Genome features of 11 cpDNAs constructed in this study.

Family	Species	Accession Number	Size (bp)	LSC Length (bp)	SSC Length (bp)	IR Length (bp)	AT Content	Core Genes PCGs/rRNAs
Bacillariaceae	*Nitzschia ovalis*	OK505007	140,703	67,862	45,741	13,760	68.7%	127/6
Bacillariaceae	*Nitzschia traheaformis*	OK505008	166,822	73,101	68,919	12,611	69.4%	129/6
Bacillariaceae	*Cylindrotheca* sp.	OK505009	188,592	98,331	61,479	14,601	70.0%	137/6
Bacillariaceae	*Cylindrotheca* sp.	OK505010	178,349	81,389	66,456	15,462	69.9%	134/6
Bacillariaceae	*Bacillaria paxillifer*	OK505012	119,449	63,955	41,892	7011	70.0%	129/6
Bacillariaceae	*Psammodictyon constrictum*	OK505011	186,015	90,740	73,153	11,271	69.2%	127/6
Naviculaceae	*Haslea tsukamotoi*	OL415006	136,784	70,894	49,194	8348	68.6%	127/6
Naviculaceae	*Haslea tsukamotoi*	OL415007	136,746	70,012	50,048	8343	68.6%	128/6
Naviculaceae	*Haslea avium*	OL415004	137,691	70,273	49,194	9112	68.7%	128/6
Naviculaceae	*Navicula arenaria*	OL415005	147,331	80,134	47,699	9749	69.3%	127/6
Pleurosigma	*Pleurosigma* sp.	OL415008	174,382	96,638	60,400	8672	70.4%	121/6

## Data Availability

All raw sequencing reads in this study were deposited to the National Center for Biotechnology Information under BioProject PRJNA746723. The datasets of chloroplast genome presented in this study can be found in online repositories (https://www.ncbi.nlm.nih.gov/, accessed on 1 July 2022). The accession number(s) can be found below: OK505007, OK505008, OK505009, OK505010, OK505011, OK505012, OL415004, OL415005, OL415006, OL415007, OL415008.
